# Personal

**Published:** 1895-09

**Authors:** 


### Personal.

Dr. Eugene Smith, professor of ophthalmology in Detroit Medical College, together with his family, has been spending the summer on his farm at Athol Springs, N. Y. Mrs. Smith and the children have returned to Detroit and Dr. Smith will soon follow. They will be greatly missed during the remainder of the season by the Lakeshore summer colony.

---

Dr. Rufus B. Hall, of Cincinnati, has taken advantage of the vacation season to renovate his private hospital for women, located in that charming suburb, Walnut Hills. This institution is one of the completest of its kind and has a well appointed operating room that is the pride of its owner.

